# Herd-level associations between the proportion of elevated prepartum nonesterified fatty acid concentrations and postpartum diseases, reproduction, or culling on dairy farms

**DOI:** 10.3168/jdsc.2023-0510

**Published:** 2024-01-15

**Authors:** J. Denis-Robichaud, I. Nicola, H. Chupin, J.-P. Roy, S. Buczinski, V. Fauteux, N. Picard-Hagen, R. Cue, J. Dubuc

**Affiliations:** 1 Independent researcher, Amqui, Québec, Canada G5J 2N5; 2 Faculté de Médecine Vétérinaire, Université de Montréal, Saint-Hyacinthe, Québec, Canada J2S 2M2; 3 TOXALIM, Université de Toulouse, INRAE, ENVT, Toulouse, France 31300; 4 Department of Animal Science, McGill University, Ste-Anne-de-Bellevue, Québec, Canada H9X 3V9

## Abstract

The objectives of this herd-level prospective observational cohort study were to describe the proportion of cows with elevated prepartum nonesterified fatty acid concentrations (PropElevNEFA) in dairy herds and to assess the herd-level associations between PropElevNEFA and postpartum diseases, reproductive performance, and culling. From November 2018 to December 2020, a convenience sample of 49 herds was enrolled in this study. Blood sampling (16 to 29 cows per herd) was performed during the week before and during the 2 wk following calving to quantify the concentration of nonesterified fatty acids (NEFA) and β-hydroxybutyrate acids (BHBA), respectively. Elevated NEFA was defined as ≥280 µmol/L and hyperketonemia as BHBA ≥1.4 mmol/L. Retained placenta, metritis, purulent vaginal discharge, endometritis, and mastitis were diagnosed on-farm following standardized definitions, and success at first artificial insemination (AI) and culling events were recorded. The associations between PropElevNEFA and each individual disease, success at first AI, and culling were evaluated using Bayesian aggregated binomial regression models with weakly informative priors, from the which odds ratio (OR) and the 95% credible intervals (BCI) were obtained. A total of 981 cows were included in the statistical analyses representing 16 to 29 (median = 19) cows per herd. Cows were enrolled in the prepartum period of their first to tenth (median = third) lactation, and 41% of them had an elevated prepartum NEFA concentration. At the herd level, PropElevNEFA varied between 11% and 78% (median = 39%). The odds of metritis (OR = 1.37, 95% BCI = 1.13–1.67) increased for every 10-point increase in PropElevNEFA, whereas the odds of success at first AI decreased (OR = 0.69, 95% BCI = 0.59–0.80). The PropElevNEFA was not associated with the other tested diseases or culling. Our results suggest that the herd-level proportion of cows having elevated prepartum NEFA concentrations is associated with metritis and poor success at first AI in dairy herds.

## Graphical Abstract

**Figure:**
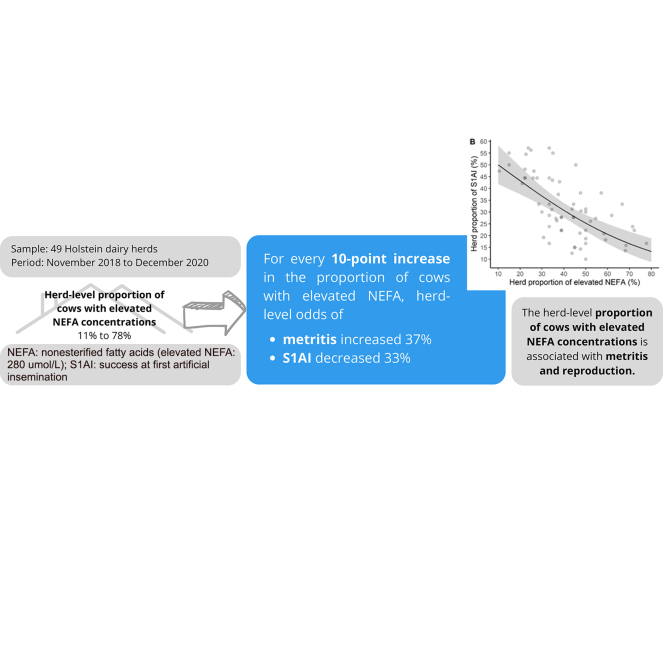

At the individual cow level, the prepartum nonesterified fatty acid (**NEFA**) concentration, generally tested 1 to 14 d prepartum, is often used as an early indicator of subsequent health, reproduction, and milk production outcomes (Dubuc et al., 2010a; Ospina et al., 2010a; Chapinal et al., 2011). As such, various thresholds (from ≥170 to 370 µmol/L) were reported to be associated with detrimental health and reproductive outcomes (Ospina et al., 2010a; Kerwin et al., 2022a; Nicola et al., 2022). At the herd level, a study conducted in 60 freestall herds from the northeast of the United States reported that herds having ≥15% of cows with elevated prepartum NEFA concentrations (≥270 µmol/L) were more likely to have an increased incidence of metabolic diseases as well as a poorer herd pregnancy rate and milk production (Ospina et al., 2010b). More recently, a similar study conducted in 72 herds from the same area reported that herds having ≥30% of multiparous cows (tested before their second lactation or greater) with elevated prepartum NEFA (≥170 µmol/L) were more likely to have an increased incidence of metabolic diseases, and that herds having ≥15% of multiparous cows or ≥40% of primiparous cows (tested before their first lactation) with elevated prepartum NEFA concentrations (≥170 µmol/L) were more likely to have a decreased pregnancy risk at first service (Kerwin et al., 2022a). These herd-level study results provide benchmarks to dairy farmers and advisors to implement surveillance strategies for assessing the herd energy balance status during the peripartum period.

In an Eastern Canadian context (province of Québec, Canada) where most herds are housed in tiestall barns and herd size is smaller than that in the Northeastern United States, it is relevant to validate if these previously reported objectives apply. A better understanding of the herd-level associations between the proportion of cows having elevated NEFA concentrations (**PropElevNEFA**) and subsequent diseases, reproduction, and culling in such a herd population would be useful to farmers and advisors. Our study objectives were (1) to quantify PropElevNEFA in commercial dairy herds from Eastern Canada and (2) to assess its association, at the herd level, with postpartum (**PP**) diseases, reproductive performance, and culling. Our hypothesis was that there are associations between PropElevNEFA and subsequent PP outcomes.

A herd-level observational prospective cohort study was conducted in commercial dairy herds that were clients of the Bovine Ambulatory Clinic of the Université de Montréal (St-Hyacinthe, QC, Canada). Of this overall herd population (n = 110), participating farms were selected by convenience by veterinarians from the clinic. The inclusion criteria were having Holstein cows, being located within a 1-h drive from Saint-Hyacinthe (QC, Canada), and participating in a regular herd health veterinary medicine program. The project was approved by the Animal Use Ethics Committee of the Université de Montréal (Rech-2059). Data collection was performed from November 2018 to December 2020. Reporting of this study was conducted using the STROBE-VET Statement checklist (O'Connor et al., 2016).

The unit of interest for the present study was the herd. A sample size of 48 herds (24 herds with a high PropElevNEFA and 24 herds with a low one) was estimated based on an estimated difference in herd-level outcome proportion of 25 percentage points (10% vs. 35%), and accounting for a variance value of 900 (30^2^), a confidence of 95%, and a power of 80% (Dohoo et al., 2009). In each recruited herd, we enrolled a minimum of 16 cows (Oetzel, 2004).

Each herd was visited every 2 wk by a veterinarian and an animal health technician. Cows were enrolled 35 (±7) d before their expected calving date. Blood samples were collected using tubes without anticoagulant (Vacutainer, BD and Co.) every 2 wk until calving occurred. These tubes were centrifuged at 1,750 × *g* for 10 min at 20°C and the serum was harvested. For each cow, the serum sample collected between 1 and 14 d before the real calving date was submitted to the Centre de Diagnostic Vétérinaire of the Université de Montréal (St-Hyacinthe, QC, Canada) to quantify the NEFA concentrations using a Beckman DxC 600 automatic analyzer (Beckman Coulter Corp.) with reagent supplied by Randox Laboratories Ltd. The analytical sensitivity of this assay was 0.1 mmol/L, and the inter- and intra-assay coefficients of variation were 3.7% and 3.9%, respectively. Blood was also sampled for each cow once during the first 2 wk PP and was analyzed cow-side for BHBA concentration using a handheld device (Precision Xtra, Abbott Diabetes Care). The analytical sensitivity of this test was 0.3 mmol/L, and the inter- and intra-assay coefficients of variation were 5.1% and 4.8%, respectively. At 37 (±7) DIM, vaginal discharge was examined using a Metricheck device (Simcro Tech Ltd.) and scored (0: absence of discharge, 1: clear mucus, 2: some flecks of purulent material in the mucus, 3: mucopurulent discharge with 50% of purulent material, 4: mucopurulent discharge with more than 50% of purulent material, or 5: fetid red-brown watery discharge; McDougall et al., 2007). A cytological endometrial sample was collected using a cytobrush (Kasimanickam et al., 2004) and tested for leukocyte esterase via a standardized technique (Denis-Robichaud and Dubuc, 2015). Retained placenta (**RP**), metritis, and mastitis were diagnosed by the farmers following standardized definitions. Farmers' recorded diseases, inseminations, pregnancy status, and culling events were collected every 2 wk during herd health veterinary visits. For logistical reasons, it was possible to blind farmers from the NEFA results but not from the PP disease diagnosis. Diseased cows were treated according to farm standard operating procedures.

We classified cows as having an elevated prepartum NEFA concentration if they had a value ≥280 µmol/L (Nicola et al., 2022). Hyperketonemia was defined as a BHBA concentration ≥1.4 mmol/L (Iwersen et al., 2009), purulent vaginal discharge (**PVD**) was defined as a vaginal discharge score ≥3 (Dubuc et al., 2010b), and endometritis was defined as a leukocyte esterase score ≥1 (Denis-Robichaud and Dubuc, 2015). Retained placenta was defined as the expulsion of the fetal membranes >24 h after parturition (Eiler and Fecteau, 2007), metritis was defined as the presence of red-brown fetid vaginal discharge with hyperthermia (≥39.5°C) and anorexia in the first 20 d PP (Sheldon et al., 2006), and mastitis was defined as the modification of the milk aspect alone or in combination with swelling of the quarter and anorexia, hyperthermia, or both (Ruegg and Erskine, 2020) in the first 30 d PP.

We performed all statistical analyses using R (version 4.2.2) with the R Studio interface (version 2021.09.0). We computed descriptive analyses at cow and herd levels. For the herd-level analyses, herds that had more than 5 missing values for a specific outcome variable were excluded from analyses for this variable. To evaluate the associations between PropElevNEFA (as a continuous variable) and each outcome, we used Bayesian aggregated binomial regression models, where the outcome was the number of cows with a given condition (e.g., success at first AI, culling event, or health condition) divided by the number of sampled cows in a herd or group.

Farm characteristics (feeding, milking, and housing system, as well as herd size and season) were considered as potential confounders and included if they modified the model estimate by more than 10% (Maldonado and Greenland, 1993). For all models, we used R as the interface to the programming language Stan (brms package; Bürkner, 2017). Weakly informative priors were used (McElreath, 2020). Priors for the regression coefficients and intercepts were normal distributions centered on 0 with a variance of 1. The models were generated using 3 chains with a length of 3,000, in which the first 1,000 iterations were used as warm-up (Homan and Gelman, 2014). Convergence was monitored by visual inspection of trace plots of variance components and density plots, and by obtaining effective sample sizes (**ESS**). An ESS of 1,000 or greater was considered sufficient for reaching convergence (Bürkner, 2017). Model fit was assessed with leave-one-out cross-validation using a moment matching algorithm if the Pareto k estimates were >0.7 (Vehtari et al., 2017). Results are presented as odds ratio (**OR**) for each 10-point change in PropElevNEFA with 95% Bayesian credibility intervals (**BCI**). We plotted associations for which the OR 95% BCI did not include 1.

A total of 981 cows from 49 herds were included in the statistical analyses. In each of these herds, 16 to 29 (median = 19) cows were sampled. For endometritis and hyperketonemia, some herds were excluded from data analysis (n = 22 and 2, respectively), because data collection was not systematic (logistical reasons). The 49 enrolled herds had between 30 and 300 (median = 80) milking cows housed in freestall (n = 12; 24%) or tiestall (n = 37; 76%) barns. In these herds, cows were either component-fed (n = 13; 27%) or TMR-fed (n = 36; 73%), and milking was done in stalls (n = 28; 57%), in a milking parlor (n = 17; 35%), or with automated milking systems (n = 4; 8%). The distributions of herd proportion of diseases, success at first AI, and culling are presented in Table 1.

**Table 1:** Descriptive analyses of herd-level proportion for postpartum diseases, success at first AI, and culling in 49 dairy herds^1^

Item	n		Descriptive analysis (proportion of)					Crude association		Adjusted association	
	Herds	Cows	Minimum	Q1	Q2	Q3	Maximum	OR	95% BCI	OR	95% BCI
Elevated NEFA^2^	49	981	10.5	33.3	38.9	50.0	77.8	NA	NA	NA	NA
RP	49	981	0	5.3	9.5	16.7	26.1	1.11	0.91–1.36	1.10^3^	0.87–1.37
Metritis	49	953	0	4.8	11.1	19.0	41.7	1.29	1.07–1.54	1.37^4^	1.13–1.67
PVD	49	949	0	4.5	7.1	12.5	47.1	0.86	0.69–1.08	0.91^4^	0.72–1.14
Endometritis	27	453	0	17.2	31.3	41.1	87.5	1.08	0.91–1.29	1.21^5^	0.98–1.50
Success at first AI	49	949	11.1	21.7	29.4	39.1	64.3	0.67	0.57–0.78	0.69^6^	0.59–0.80
Hyperketonemia	47	924	0	12.5	20.8	29.0	50.0	1.04	0.88–1.23	1.01^4^	0.85–1.20
DA	49	953	0	3.7	8.3	11.8	20.0	1.10	0.86–1.37	1.08^7^	0.84–1.38
Mastitis	49	953	0	4.8	6.3	14.3	40.0	1.19	0.97–1.47	1.15^8^	0.93–1.41
Culling	49	981	0	0	0	5.6	13.6	1.06	0.75–1.47	1.10^4^	0.77–1.55

1 The association with the proportion of cows having elevated prepartum nonesterified fatty acid (NEFA) concentrations (for every 10-point increase) is also presented. Odds ratios (OR) and 95% credible intervals (BCI) were calculated using Bayesian aggregated binomial regression models. RP = retained placenta; PVD = purulent vaginal discharge; DA = displaced abomasum; NA = not applicable; Q1 = quartile 1; Q2 = quartile 2 (median); Q3 = quartile 3.

2 ≥280 μmol/L.

3 Adjusted for season, feeding, and milking system.

4 Adjusted for season and feeding system.

5 Adjusted for season, herd size, housing, feeding, and milking system.

6 Adjusted for season.

7 Adjusted for season, herd size, and feeding system.

8 Adjusted for herd size.

Cows were enrolled in the prepartum period before their first to tenth (median = third) lactation. Individual prepartum NEFA samples were collected 1 to 14 (median = 7) d prepartum and their values ranged from 61 to 1,606 µmol/L (median = 243 µmol/L), with 41% (n = 405) of overall cows having an elevated prepartum NEFA concentration (≥280 µmol/L). At the herd level, PropElevNEFA varied between 11% and 78% (median = 39; Table 1), and 96% of the herds were over the threshold of 15% (Ospina et al., 2010b). Herd-level associations between PropElevNEFA and metritis, as well as between PropElevNEFA and success at first AI, were found (Figure 1). Specifically, for every 10-point increase in the PropElevNEFA, the odds of metritis at the herd level increased by 37%, whereas the odds of success at first AI decreased by 31% (Table 1). The odds of RP, PVD, endometritis, hyperketonemia, displaced abomasum, mastitis, and culling were not associated with the herd PropElevNEFA (Table 1).

**Figure 1:**
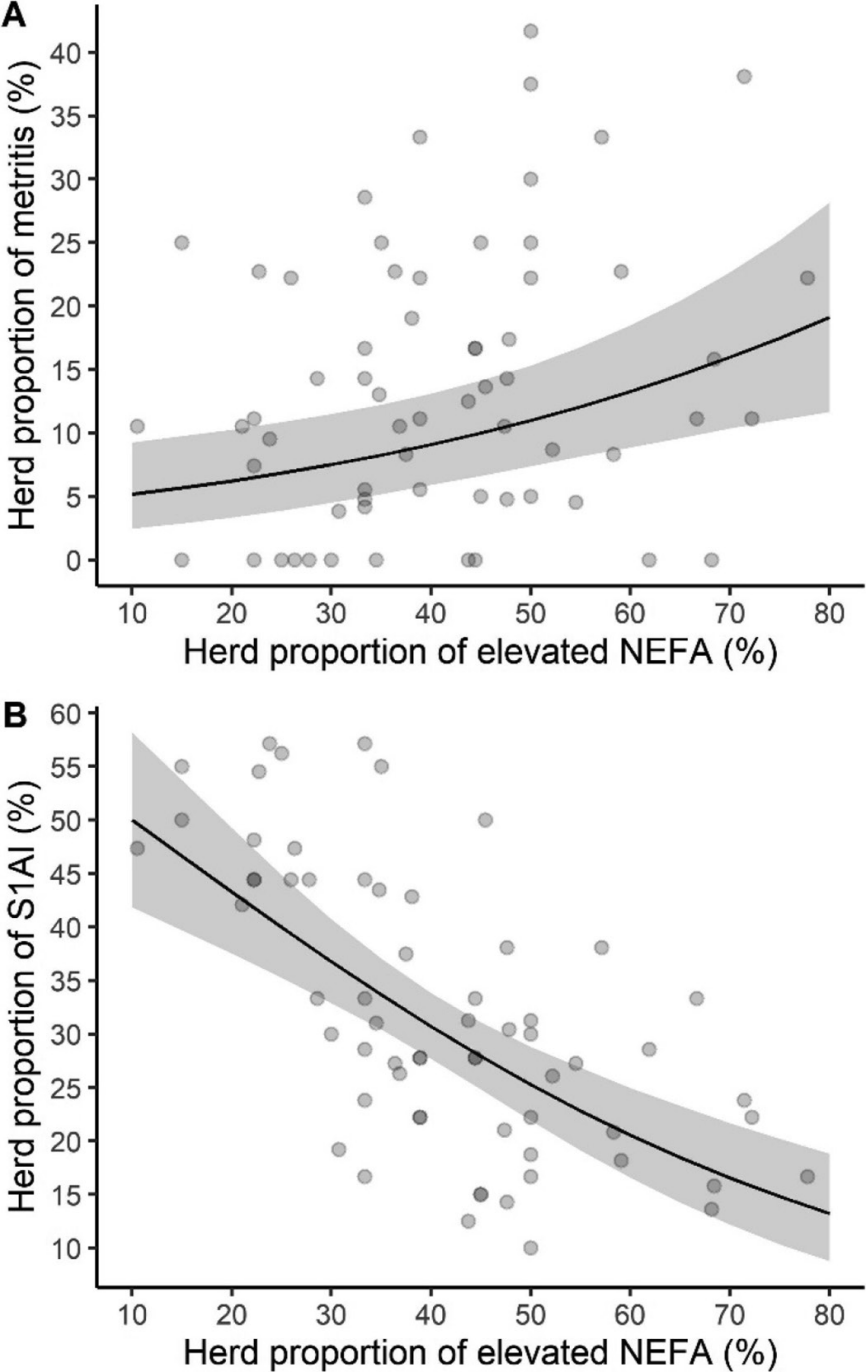
Posterior predictions (line) and 95% credible intervals (ribbon) from Bayesian aggregated binomial regression models evaluating the association between the herd-level proportion of cows having elevated nonesterified fatty acid concentrations (NEFA ≥280 µmol/L) and (A) metritis or (B) success at first AI (S1AI). Models were built using data (scatterplot) from 49 dairy herds.

Overall, our results demonstrate that PropElevNEFA was associated with an increased proportion of cows having metritis, as well as with a decreased proportion of success at first AI. Interestingly, almost all our participating herds had a PropElevNEFA greater than the herd-level threshold suggested by a previous study (Ospina et al., 2010b). We believe this could be caused by our source population, which might be different from that of the studies conducted in the Northeastern United States and previously published. It is also possible that a selection bias was created when recruiting herds. The same rationale applies to the herd selection process used by previously published studies (Ospina et al., 2010a,b; Kerwin et al., 2022a); recruiting 49 to 72 herds to conduct studies is labor intensive for researchers but remains an overall small sampling of a regional dairy herd population. This situation likely influenced the distributions of PropElevNEFA and PP diseases, which seem to be higher in our study than previously described elsewhere (Dubuc and Denis-Robichaud, 2017; Kerwin et al., 2022a). Future extensive studies using a randomized selection of herds within the general herd population are needed to better understand our findings.

The association we found between PropElevNEFA and the decreased odds of success at first AI is consistent with a decreased pregnancy rate reported elsewhere (Ospina et al., 2010b; Kerwin et al., 2022a). This finding is consistent with the negative association between prepartum NEFA and reproduction at the cow level (Giuliodori et al., 2013; Denis-Robichaud and Dubuc, 2015; Machado et al., 2020) and the herd level (Dubuc and Denis-Robichaud, 2017). These findings are supported by multiple studies demonstrating the negative effects of elevated NEFA concentrations on immune functions (reviewed by Lacasse et al., 2018). Interestingly, we did not find any associations between PropElevNEFA and some infectious diseases such as PVD, endometritis, or mastitis. It was not logistically and ethically feasible to prevent farmers from treating their diseased animals, and this situation could have biased our measures of association between PropElevNEFA and reproductive tract diseases (PVD or endometritis). Considering that cows having metritis early in the postpartum period are more likely to develop PVD or endometritis later on (Dubuc et al., 2010a), treating the metritic cows with antibiotics might have reduced the occurrence of PVD and endometritis. It is also possible that our herd sample size did not allow us to capture associations of smaller magnitude than anticipated during the sample size estimation procedure. Although recruiting 49 herds might not be enough, it implies a lot of logistical work coordination and it would have been difficult in our context to recruit a greater number of herds. These limitations should be kept in mind when making inferences from our results.

Many studies have reported the relationships between PropElevNEFA and hyperketonemia or displaced abomasum at the cow (Ospina et al., 2010a; McArt et al., 2013; Kerwin et al., 2022b) and herd levels (Ospina et al., 2010b; Kerwin et al., 2022a). However, this was not the case in our study. Even if the NEFA and BHBA concentrations are both used as markers of energy balance during the transition period, their correlation throughout the peripartum period remains weak (McCarthy et al., 2015). It remains unclear why our results are not consistent with previously published literature, and further studies are necessary.

In conclusion, our results demonstrate a herd-level relationship between PropElevNEFA and metritis as well as between PropElevNEFA and success at first AI. These results help to understand the negative impacts of having a high PropElevNEFA on dairy farms.
